# Supplementary material for: The MoveStrong program for promoting balance and functional strength training and adequate protein intake in pre-frail older adults: A pilot randomized controlled trial
Source: PLoS One. 2021 Sep 24;16(9):e0257742. doi: 10.1371/journal.pone.0257742 (PMC8462677; doi:10.1371/journal.pone.0257742)
Supplement: S2 Appendix — (PDF) [file pone.0257742.s002.pdf]

A new model to deliver strength and balance training and nutrition education for pre-frail/frail older adults (MoveStrong): A pilot randomized controlled trial

ABSTRACT

Background: There is strong evidence certain types of exercise can improve physical functioning and disability outcomes in older individuals with frailty. However, many older adults do not meet the physical activity guidelines for strength training. Novel concepts and models with the potential for large scale implementation and long-term adherence to strength exercise are urgently needed for frail older adults.

Objective: The purpose of this study will be to assess the feasibility of implementing community-based workshops to teach older adults about functional strength and balance training and methods to increase protein intake.

Design: Closed cohort stepped wedge design.

Setting: Northern (rural) and Southern Ontario sites in Canada

Participants: We will recruit 40 older adults (≥60 years) considered pre-frail/frail as measured by the FRAIL Scale with at least one chronic condition who are not currently engaging in a regular strength training.

Intervention: The MoveStrong study is an eight-week exercise and nutrition education program that includes a kinesiologist-led twice-weekly functional strength and balance program, and two seminars delivered by a dietitian to promote adequate protein intake.

Measurements: The primary outcome is feasibility of implementation defined by recruitment, retention, and adherence rates. Secondary outcomes include frailty indicators, mobility and balance measures, health-related quality of life, dietary protein intake, adverse events, resource costs, and

participant and provider (i.e., kinesiologist) experience with the program. We will analyse feasibility using descriptive statistics based on estimates with 95% confidence intervals. Secondary outcomes will be evaluated in exploratory intention-to-treat and per protocol analyses via independent student *t* tests or logistic regression. Content analysis will be used to identify facilitators or barriers to implementation.

Conclusions: Our goal is to test a scalable model to enable pre-frail/frail older adults to participate in functional strength and balance training and to consume an adequate amount of protein. The current study will inform the feasibility of a larger pragmatic trial in other diverse settings.

Registration: This trial was registered on August 6th, 2019 in ClinicalTrials.gov under the registration identifier NCT04037436.

INTRODUCTION

With age, there are changes in body composition including atrophy of the skeletal muscle, beginning around age 25 and accelerating after the age of 50 (1,2). Sufficient muscle strength is necessary to provide a reservoir of protein for the immune system, prevent falls and disability, and perform activities of daily living such as getting up from a chair. When age-related loss of muscle mass decreases beyond a defined threshold, this is termed sarcopenia, "a progressive loss of skeletal mass and strength that increases the risk of adverse outcomes such as physical disability, poor quality of life, and frailty" (1,3). Between 1% to 29% of community-dwelling older adults have sarcopenia, and this condition has direct consequences on personal, social, and healthcare costs (1,4). Sarcopenia is a major cause of disability and frailty in the elderly and the physical features of frailty include weakness, slowness, unintended weight loss, and low physical activity (5).

There is strong evidence that regular physical activity can improve muscle outcomes in older adults (4,6–8). There is also emerging data for significant psychological and cognitive benefits accrued from regular exercise (8). A recent meta-analysis reported that exercise interventions significantly improve some aspects of muscle strength and mass, and physical functioning and mobility outcomes in older adults with sarcopenia (9). Specifically, resistance training alone, performed between 3 to 18 months, improved muscle mass and strength, and physical performance variables such as chair rise, stair climb, and the 12-minute-walk-test in older adults with sarcopenia (4). Progressive resistance training performed two to three times per week at a high intensity result in moderate to large improvements in gait speed, getting out of a chair, and muscle strength (10). Further, a recent Cochrane review reported that balance and functional exercises reduced the rate of falls by 24% in community dwelling older adults (11), and balance and functional exercises in

combination with resistance training could potentially reduce the rate of falls by more than 30% (11).

The benefits accrued from exercise are evident, but over 75% of Canadian adults 18 years and older are not meeting the physical activity guidelines, and it gets worse with age, with 88% of adults over 65 not meeting the guidelines (12). Thus, the biggest challenge is not a lack of evidence that exercise is beneficial, but the absence of effective, sustainable real-world implementation exercise models, especially for older adults with chronic conditions. Using what we learned from previous work on pilot trials (13), we collaborated with the YMCA, Community Support Connections, Osteoporosis Canada, and patient advocates to co-create MoveStrong – a model of service delivery that adapts functional strength and balance training with attention to nutrition to support movements performed during activities of daily living in pre-frail/frail older adults.

Objectives

The pilot study will assess the feasibility, fidelity and adaptability of the MoveStrong model in diverse settings and responsiveness of frailty outcomes. The primary objectives will be to:

1. Evaluate the number of participants recruited to participate at the start of the study;
2. Determine retention rates at the end of the study; and
3. Calculate adherence rates to the MoveStrong program.

Secondary objectives will determine participants' and providers' experience with MoveStrong, adaptations to the model, the cost relative to the benefit, the short-term responsiveness of frailty indicators, protein intake, health-related quality of life, and if the exercise and the nutrition behaviour are maintained.

METHODS:

The protocol was drafted in accordance with the SPIRIT 2013 checklist (https://www.spirit-statement.org/) and the TIDieR checklist (see Table 1).

Trial design and Timeline

This is an 8-week pilot single-blinded, multicentre, randomized controlled trial (RCT). We will use a closed cohort stepped wedge design at four sites across Ontario. In a stepped wedge study, the design provides each site exposure to the intervention but not at the same point in time. Before the program begins, all sites are randomized to start at one time point. Then, at regular intervals (the "steps") one site will cross from the control to the intervention (see Figure 1) (14). This process continues until all sites have been exposed the MoveStrong program.

Study Setting

One Northern and three Southern Ontario sites were chosen to ensure diversity in city population, structure, and services. We will evaluate the MoveStrong program at three distinct settings: retirement home, YMCA, and a family health team. The MoveStrong program will be implemented and delivered at a kinesiologist-led clinic partnered with the City of Lakes Family Health Team (Sudbury), Arbour Trails (retirement home and independent living, Guelph), Village of Winston Park (retirement home and independent living, Kitchener), and two of the YMCA's of Cambridge and Kitchener-Waterloo (CKW YMCA, each YMCA is part of one site). The Sudbury site is located in Northern Ontario, while the other three are in Southern Ontario.

Eligibility Criteria

Residents are eligible to participate in the study if they: speak English or attend with a translator; ≥ 60 years of age; have a FRAIL Scale score ≥1; and have ≥1 primary care diagnosed chronic condition [e.g., diabetes, obesity, cancer (other than minor skin cancer), chronic lung disease, cardiovascular disease, congestive heart failure, hypertension, osteoporosis, arthritis, stroke,

or kidney disease]. Participants will be encouraged to attend with a caregiver/friend for social or physical support, and the caregiver/friend can choose to complete the screening and assessment process if eligible. Residents cannot participate in the study if they: are currently doing a similar resistance exercise ≥2x/week; are receiving palliative care; cannot perform basic activities of daily living; have severe cognitive impairment (e.g., unable to follow two-step commands); travelling >1 week during the MoveStrong program; and have absolute exercise contraindications. Absolute exercise contraindication will be assessed using the American College of Sports Medicine guidelines and they must seek physician approval before exercising. In case of medical uncertainty, the study's medical advisor will be consulted. If the medical advisor is uncertain, we will ask the potential participant to contact their physician to provide consent.

Intervention

Exercise Program: MoveStrong is designed to provide a scalable framework for exercise professionals to tailor fundamental strength training exercises for older adults of varying abilities, using minimal equipment. The exercises are aligned with functional movements to promote personal relevance; jump, step-ups, reach, squat, pull, lift and carry, and push. Seated exercises are provided only for participants who cannot perform the lowest level of difficulty (Level 1) with or without external support (e.g., walker, cane, wall, table). Exercises are informed by the GLA:D program for arthritis (15), BoneFit™ (16), and meta-analyses on resistance exercise and fall prevention (7,10,17–19). Each participant will have a 1:1 session with a kinesiologist (not blind to cluster allocation) who will select a starting level and variations for each functional movement, intensity, and the number of repetitions and sets. Then, participants will attend a kinesiologist-led exercise workshop (5 attendees, 1:5 kinesiologist to participant ratio) twice a week for 8 weeks. The exercise program starts with a warm up (5-7 minutes) that include a reactive stepping game

(http://clockyourself.com.au) or other balance and agility activities. Participants then complete the MoveStrong exercises where they perform 2-3 sets of 3-8 repetitions of each exercise with time under tension per repetition of 4:0:2 seconds for eccentric:isometric:concentric phases (Table 2). During the first two weeks, the focus is on form rather than on intensity. Exercise difficulty, resistance used, or volume (up to 3 sets, up to 8 reps) is progressed over time, with a target intensity of < 8 repetitions maximum. At the end of each session is a 10-minute group discussion where participants select an "Exercise of the Week" to plan when and where to practice it at home, or in a setting of choice. Each site will receive a standardized toolkit with materials for participant workbooks and a trainer manual. The manual provides guidance on how to deliver the workshop, select and progress exercises, adapt exercises for common impairments, cueing tips, and discussion topics. Participant workbooks will be assembled to include pictures of each exercise (i.e., the variation of each movement selected for them) with instructions so they can take home to practice and exercise logs and planning worksheets.

Nutrition Education: Participants receive an education booklet and will participate in two dietitian-led group seminars that discuss strategies to increase protein intake. Presentations topics will consider the cost of preparing high protein foods and the ability of retirement home residents to alter their diet when food is provided, a guide how and why to spread protein intake through the day, how much protein is in their usual diet, low cost options, easy-to-consume protein-rich snacks with minimal preparation, high quality protein supplements (e.g., rapidly digested, high leucine like whey), and how to prioritize high-protein choices in retirement home menus or restaurants. Other activities include sampling of protein-rich snacks. Seminars will occur at weeks 2 and 5 to allow time to review material, revisit topics, and address questions. We will promote a protein intake

greater than 1.5 g/kg of body weight/day or 20-30g of protein/meal. In the pilot study, seminars will be delivered in person for all sites. The dietitian will not be blind to cluster allocation.

Outcomes

The primary research question is feasibility of implementation, defined by recruitment (number of participants recruited at the end of rollout), retention (number retained at post-rollout end), and adherence (percentage of exercise sessions completed). Our criteria for success are to recruit 10 participants at each of the four sites (40 total), retention of 90% at post-rollout end, and adherence of ≥70% (13,20). Table 3 lists all variables, hypotheses, outcomes and methods of analysis. We will also measure the following secondary outcomes:

a) *Frailty indicators:* Fried Frailty Index components guided the selection of frailty indicators (21). Height will be measured via stadiometer or wall mounted measuring tape. We will measure change in body weight with a scale, walking speed via the 10-metre walk test (22), fatigue with the Center for Epidemiologic Studies Depression Scale-fatigue questions (CES-D) Depression Scale (23), and physical activity levels using a variation of the questions used by Exercise is Medicine. Only two questions on the CES-D will be used: 1) I felt that everything I did was an effort and 2) I could not get going. To assess handgrip strength, we will follow the 2016 National Institute for Health Research Southampton protocol and use a digital Jamar Hand Dynamometer to measure isometric grip force in both hands (24,25). To test functional leg strength, we will use the 30-second chair stand test that requires a chair with a straight back against a wall, 17 inch high, without armrests and rubber tips on the legs (26). Participants will be asked to sit in the middle of the chair, place their hands on the opposite shoulder crossed at the wrist, feet flat on the floor with a straight back (26). When the research assistant says, “go”, the participant will rise to a full standing position and then sit back down again as many times as possible for 30

seconds (26). Foot clearance is an important function in everyday life and the ability to accomplish this in different directions is essential when reacting to stimuli in the real world (i.e., navigating a busy street or walking on uneven pavement) (27). The Four Square Step Test incorporates rapid stepping whilst changing direction; a square is formed using four 90 cm long canes resting flat on the floor and the participant will step in each square as fast as possible (28). Each square is labelled 1 to 4. The participant will start in square 1 facing square 2 and then step forward into square 2, sideways to square 3, backward to square 4, sideways to square 1, sideways to square 4, forward to square 3, sideways to square 2, and backward to square 1 (28).

b) *Quality of life and resource use:* The EuroQol Group 5 Dimension 5 Level (EQ-5D 5L) questionnaire is a multi-attribute health related quality of life tool that we will use to achieve a quality-adjusted life year (QALY) estimate per intervention (29). We will use a questionnaire to assess healthcare utilization, including direct medical (e.g., personnel, hospitalization, medications, rehabilitation, tests), direct non-medical (e.g., out of pocket expenses, transportation), and indirect resources used over the last 6 weeks. Multiplying resources collected by jurisdictional unit costs will determine the total cost per exercise program.

c) *Dietary energy/protein intake:* We will use the Automated Self-Administered 24-Hour (ASA24®) Dietary Assessment Tool (epi.grants.cancer.gov/asa24/) to conduct interviewer administered diet recalls for 2 weekdays and 1 weekend day. Nutrient analysis is automated and will be used to quantify and compare protein and energy intakes at baseline (week 1) and at follow-up (week 36).

d) *Participant and provider experience and satisfaction, adaptations, fidelity:* We will use a semi-structured interview guide to conduct exit interviews with each participant and the kinesiologists (see Table 4 for examples). Interviews will be audio-recorded and transcribed verbatim. One

researcher will perform content analyses to describe participant and provider experience and satisfaction, adaptations, and learning needs. Analyses will be verified by another research through member checking. Kinesiologists and staff will be given a spreadsheet to record any protocol adaptations, challenges and successes to inform a future trial. We will observe a MoveStrong session at each site and evaluate fidelity via fidelity checklist.

e) *Adverse Events:* We will ask participants to report adverse events such as falls, fractures, muscle pain, etc., using Health Canada definitions (30). Any major adverse events (e.g., fracture, death, hospitalization event) will be reported to the principal investigator and to a Data Safety Monitoring Committee made up of three arms' length members (a physician, a physical therapist and a biostatistician).

Recruitment

We aim to recruit all 40 participants prior to randomizing the sites. Participants will be recruited from local primary care practices, retirement homes, and via advertisement in the local community. We will also use social media (Facebook and Twitter) to advertise the MoveStrong study and share our recruitment posters with local contacts. A research assistant from the University of Waterloo and two nurse practitioners from the Sudbury site will recruit participants.

Sample size

Sim and Lewis 2012 (31) and Julious 2005 (32) recommend at least 50 participants or 12 individuals per group, respectively, for a pilot study. However, we selected a recruitment rate of 10 participants at each site (four sites) because of the proposed class ratio of one instructor to five participants. Recruiting 10 participants will also allow us to observe feasibility of delivery of two full nutrition sessions of five people each. We will allow sites to over-recruit by two people if they are able to, and allow 6 participants per exercise class.

Randomization and Allocation

After 40 participants have been recruited and provide informed consent, a biostatistician will develop a computer-generated randomization sequence at St. Joseph's Healthcare in Hamilton to randomize sites to implement MoveStrong at one of four start times, each three weeks apart. A co-investigator at the University of British Columbia will keep the randomization sequence concealed and communicate it to the sites after randomization.

Blinding

A research assistant will complete all baseline assessments and will conduct an additional three assessments each six weeks apart. Follow-up assessments after randomization will be performed by assessors blinded towards site randomization. One research assistant will perform all outcome assessments at the three Southern Ontario sites, and one nurse practitioner will complete the assessments in Northern Ontario.

Data Collection and Management

Questionnaires may be completed over the phone, if it is not possible to complete them in a visit. Telephone and in-person screening data will be collected using paper records. The research assistant or an undergraduate student will then transfer the paper records into an Excel file. We will record if any assessor is unblinded as part of the feasibility evaluation. The kinesiologist at each site will use a standardized log to record adherence. Each site will be required to keep accurate and verifiable source notes relevant to each study participant and a protocol deviation log. Participants will be de-identified by assigning an ID to be used in all paper and electronic data collection. Identifiable information (i.e., full name, date of birth, site number, and contact details) will be kept separately in a secure location.

Data Safety Monitoring Committee (DSMC)

The role of the DSMC is to monitor the study processes, including any safety or ethics concerns for participants in particular. We are recording adverse events and serious adverse events that occur during the study, and will provide the DSMB with reports on serious adverse events (e.g., falls, fractures, cardiac episodes, etc.,), and on flow through the study, as well as other information the DSMB requests. The DSMB will review interim information about the above including all recruitment, randomization and completeness of outcome assessments when half of the target number of participants have completed the intervention phase. The DSMB will be provided with information in the Open report about the study protocol on the design, timeline, recruitment, randomization and data collection, the Statistical Analysis Plan, and the DSMB Charter. Information about the secondary outcomes, including adverse events will be presented in the Closed Report to the DSMB. The content of these reports is outlined in section 7. The DSMB Chair can request additional meetings.

The DSMC will review adverse events after two sites have completed half of the program. The DSMC will have unblinded access to all data. No interim analyses are planned and there are no stopping guidelines for the pilot trial.

Statistical analyses

Demographic data and outcomes will be summarized using descriptive measures such as mean and standard deviation or mean and confidence intervals for continuous variables, and count and percent for categorical variables. Recruitment, retention and adherence will be reported as a mean and standard deviation or estimates based on 95% confidence intervals (Table 3). To assess responsiveness of frailty indicators, we will calculate standardized response means, or mean change in score divided by the standard deviation of the changed scores. We will conduct exploratory analyses of secondary outcomes using linear regression, adjusted for period, time by exposure

interaction and intra-cluster correlation (SASv9.2, North Carolina), and sensitivity analyses with/without caregiver or friend participation. Clusters will be analyzed according to their randomized exposure, regardless of whether exposure was achieved at the correct time. We will compare groups using per protocol analysis and exploratory intention-to-treat analysis and we will impute missing data using multiple imputation. The criterion for statistical significance will be set at alpha ≤ 0.05. To analyse the exit interviews, we will do content analysis using NVivo version 12 Pro or higher (QSR International Pty Ltd, 2019) to identify positive or negative experiences and suggested adaptations to the program. Adverse events will be reported with descriptive statistics or estimates based on 95% confidence interval.

Ethics and Dissemination

Research Ethics Approval

The research will be conducted according to the 2014 Tri-Council Policy Statement, (http://www.pre.ethics.gc.ca/eng/policy-politique/initiatives/tcps2-eptc2/default/). The study has received approval from the University of Waterloo Integrated Research Ethics Board. Any future amendments will be submitted to the ethics board and updated in the registered clinical trials protocol.

Protocol Amendments

Protocol amendments will be submitted to the University of Waterloo ethics board and to ClinicalTrials.gov. Plans for communicating important protocol modifications such as eligibility criteria, outcomes, analyses will be supported with a letter to explain the need for these changes.

DISCUSSION

Although there is strong evidence to support the effectiveness of functional strength and balance training to improve physical performance outcomes in older adults, there is variability in how exercise programs should be implemented in clinical practice (33–35). Due to the high

relevance and socioeconomic impact of frailty in the older population, it is not surprising that a number of studies have been conducted to address frailty and its health-related consequences. Previous pragmatic trials evaluating the implementation of exercise interventions in older adults have included a home-or-facility-based exercise program, an exercise referral scheme (ERS), and physical activity counselling.

The Otago Exercise Program (OEP), a home-based exercise model, successfully reported participants reaching at least 150 minutes of moderate to vigorous physical activity per week rose from 41% to 43% in the OEP arm (36). However, its dissemination in the USA has been limited due to misconceptions about billing practices, increased paperwork for the therapist, inability to bill for telephone follow-ups, and misalignment between the structure of the OEP and Medicare documentation policies (37). Another home-based exercise model, the Lifestyle-integrated Functional Exercise (LiFE) program, teaches older adults to integrate exercise into their daily activities over five home-based visits. The program was associated with an increase in self-reported physical activity, a 31% reduction in fall rates, and improvements in balance and strength compared to the control (38). LiFE is effective, and a larger clinical trial is examining its suitability for recommended implementation on a larger scale (39). In the ERS model, a physician refers a patient to an external healthcare provider or community program and when compared to usual care, 11% (95% CI 2% to 26%) more ERS participants achieved 90 to 150 minutes of moderate intensity physical activity per week at 6 to 12 months of follow-up (40). However, it is unclear if this strategy promotes long-term adherence for frail elderly patients that may require a more tailored exercise prescription since none of the studies reported outcomes of ERS by disease-specific populations. Lastly, physical activity counselling is a model where a physician or nurse provides intensive physical counselling as part of an interdisciplinary family health team. In a number of Danish

countries, Exercise on Prescription (EoP), a type of physical activity counselling, was used in general practice in an attempt to improve physical activity levels among sedentary patients with chronic diseases. A systematic review reported participants in the EoP group increased physical activity levels by 10% compared to the control group but the majority of studies were of low quality (41). While primary care is an ideal setting to counsel older adults in need of exercise interventions, individualized exercise prescriptions may not be feasible due to cost and time restrictions.

The MoveStrong model adapts components from the ERS model (where physicians or other healthcare professionals can refer a patient to the MoveStrong program) and from the facility and home-based exercise models to help improve uptake of physical activity in pre-frail/frail older adults. The program is designed to be scalable in multiple settings, with training materials for instructors to tailor, teach, and progress functional moderate-to-high intensity strength and balance training. A unique feature of the program is the fundamental strength training exercises align with functional movements to promote personal relevance such as *step-ups* to build stair-climbing power or the *loaded carry* to transporting groceries more efficiently. Balance challenges are included for fall prevention (18,19). The MoveStrong intervention also provides a social interaction and in a qualitative study of older adults with cardiac events, factors that influenced uptake of exercise and short term adherence included social support (e.g., health professionals, family, and friends), structured classes (novel exercises, specialist staff, routine, purpose), health (belief in health benefits), and high self-efficacy (42). We will encourage participants to bring a friend/caregiver to increase social support and provide the participant a toolkit with a participant exercise book and tracking sheet, a nutrition booklet, and Therabands. Other strategies to increase future uptake and adherence included support (a reasonable staff-to-participant ratio and group meetings/discussions) and motivation (challenge and variety, goal setting and feedback) (42). For example, the

MoveStrong intervention was designed to increase uptake and adherence by providing a 1:5 kinesiologist to participant ratio and by offering exercises on a continuum of difficulty to maintain motivation. The Kinesiologist will also be provided with a trainer manual with guidance on adaptations for common impairments, cueing tips, and discussion topics to encourage the participant to exercise safely. To maximize the benefits of resistance training, the program provides two dietitian-led seminars and a booklet focused on using real food to improve protein intake. From past work, we identified meal preparation approaches with fresh ingredients are preferred over supplements. Lastly, we designed the intervention with a follow-up post-exposure to examine whether behaviours or effects are sustained after workshop cessation. There is a need to continue to develop and refine strategies to increase the uptake of exercise among pre-frail and frail older adults, and, accordingly, the proposed study protocol hopes to extend the current knowledge in this area.

The MoveStrong trial has a few strengths. The intervention incorporates behavior change techniques, such as a kinesiologist led exercise sessions and social engagement, to promote higher uptake and adherence of physical activity. It also uses a eligibility criteria consistent with a pragmatic trial (43) such that we will include a wide range of participants to meaningfully assess the feasibility of and implementation for our trial. In addition, the stepped wedge design provides the advantage that all participants will eventually receive the intervention making recruitment easier (44). A limitation of our pilot trial is the short follow-up period, which prevent us from examining the long-term maintenance of our program but this can be evaluated in a larger trial in the future. Another limitation is the concern that an intervention implemented in all clusters has not yet been proven effective in a stepped wedge design (44). However, despite this limitation we chose to this design since there is mounting evidence that exercise interventions can improve a number of health

outcomes in older adults (4,6–8). It can also be argued that there are some circumstances where the stepped wedge trial is preferable to the parallel RCT (45) since the stepped wedge design is superior both scientifically (as more data can be obtained) and ethically (since all groups can receive the intervention).

Trial Status

The study is still ongoing and we expect to complete the study by September 2020. By the time of submission, 40 participants were already enrolled in the trial and data collection started.

CONCLUSION

There is an abundance of research of the benefits of strength training for people with chronic diseases, but research has consistently shown that the majority of older adults do not meet the exercise guidelines. This non-compliance has been associated with substantial costs, including avoidable morbidity, increased hospital admissions, and nursing home admissions. The purpose of this pilot study is to test the feasibility to implement a functional exercise and nutrition education program in rural and urban communities across Ontario. The intervention includes behaviour change strategies, social interaction and education to increase protein intake to maximize the benefits of resistance exercise.

OTHER INFORMATION

Registration: This trial is registered in ClinicalTrials.gov Identifier NCTO20190401.

Funding: Funding for this project was provided by The Canadian Institutes of Health Research (CIHR-SPOR grant) [CIHR Funding Reference Number SCT-162968]. The funding agent will not provide input on the study design, collection, management, analysis, interpretation of the results or the decision to submit the report for publication.

Appendix A

	Study start-up training	Pre-rollout period	Rollout, Exposure and Maintenance																			Data cleaning, analysis, dissemination
**Time in weeks**	**1 to 8**	**9 to 16**	**17 to 18**	**19**	**20**	**21**	**22**	**23**	**24**	**25**	**26**	**27**	**28**	**29**	**30**	**31**	**32**	**33**	**34**	**35**	**36**	**37 to 52**
Site 1			x						x						x						x	
Site 2			x						x						x						x	
Site 3			x						x						x						x	
Site 4			x						x						x						x	

- Training of staff, orientation to study and materials
- During the pre-rollout period, study sites will initiate advertisement and recruitment
- Roll-out period: Intervention is rolled out every three week
- Exposure to MoveSTroNg Program
- Maintenance period: MoveSTroNg workshop is complete. Participants are encouraged to sustain behaviour change
- Repeated measurements from individuals at fixed time points, allowing assessment of period effect

Figure 1: MoveStrong study design, allocation strategy, and timeline

Table 1: Template for Intervention Description and Replication (TIDieR) Checklist for the MoveStrong Program

Item Category	Description
Brief name	MoveSTroNg: A model for delivering Strength Training and Nutrition education for older adults in Canadian communities.
Why	The purpose of this study is to investigate whether an exercise program that focuses on basic daily movements with information on healthy eating and advice on how to change behavior can help older adults improve muscle strength and physical function in a way that participants will adherence too.
What: *Materials*	1) Participant's booklets will be used during and after the intervention. The booklet contains a series of pictures and written instructions on how to preform each movement safely and with proper form;<br>2) Participant's nutrition booklets will be used during and after the intervention. The booklets contain tips and recipes complimented by pictures and visual cues on methods to increase protein intake throughout the day;<br>3) Instructor's manual will be provided to the Kinesiologist prior to the start of the program and contains information on how to run the exercise programs (e.g., equipment and set-up, how to select and teach each exercise, safety, warm-up and cool down, etc.), cueing tips, and motivational interviewing strategies to promote participant adherence.<br>4) Study manual will be provided to the outcome assessors, the Kinesiologists, the dietitian, and other individuals involved in implementing the MoveStrong program. The manual contains information about the program timeline, research forms, physical assessment forms, and adverse event reporting forms.<br>5) Equipment will be provided to all sites including Therabands (3 levels), Kettlebells (5, 10 and 15 lbs), and step-ups with modifiable levels
What: *Procedures*	The Kinesiologist will review each participant's medical history and meet with each participant one-on-one prior to the start of the program. The participant and the Kinesiologist will select one of four starting levels for each movement. There are seven functional movements (see Table 2) and each movement will be progressed as necessary.
Who: *Provided*	Exercise sessions will be delivered by a Kinesiologist with at least 1-year experience working with older adults. The nutrition sessions will be offered by an experienced dietitian.
How	After randomization, one site will start the MoveStrong program. The intervention is provided face-to-face in a group setting with 1 Kinesiologist to 5 participants.
Where	There are four sites where the program will be implemented: 1) Kinnect to Wellness (Sudbury); 2) Arbour Trails (retirement home and independent living, Guelph); 3) Village of Winston Park (retirement home and independent living, Kitchener); and 4) YMCAs of Cambridge and Kitchener.
When and how much	Frequency/Duration: 2x/week for 8 weeks, 60 to 90 minutes/session;<br>Intensity: 2-3 sets of 3-8 repetitions of each exercise with time under tension per repetition of 4:0:2 seconds for eccentric:isometric:concentric phases;
Tailoring	Irrespective of the method of delivery, individual tailoring will be constantly given due to the activities' integration in the participants' personal routine. The frequency and intensity of each movement will be determined by the participant and the kinesiologist during 1-on-1 sessions before the start of the program.
Modifications	No modifications at this time.
How well: *Planned*	A third party, who is not involved in collecting outcome data, will assess if the intervention is delivered and performed as it was intended using a Fidelity Checklist.
How well: *Actual*	Not applicable at this time.

Table 2: MoveStrong Movements with Progressions

Kinesiologist selects 1 version per movement for each participant (intensity 3-8RM, volume 2-3 sets, 3 to 8 repetitions, time under tension per rep of 4:0:2 seconds, eccentric:isometric:concentric). Progression: increase resistance, weight or volume (e.g., sets, reps), or select a harder exercise. Seated exercises are for participants who cannot perform level 1, even with a support object.

Functional movement	Seated Version[3]	Level 1	Level 2	Level 3
Jump				
Step ups				
Reach or Press				
Squat				
Pull				
Lift and carry				
Push				

[1]Resisted: use elastic tubing or bands. Weighted: weights or household objects, held close to body e.g., water bottles, 4L jugs of water, weighted grocery bags or backpack. Priority is form over intensity. [2]Include forward and backwards chaining of getting on and off floor if that is participant's goal, with or without chair or support object. [3]May consider starting with object on elevated surface.

.

Table 3: Variables, hypotheses, outcomes and methods of analysis

Variable	Hypothesis	Outcome Measures	Methods of Analysis
Primary			
**Recruitment**	We will recruit 40 individuals, 10 per site	• Number recruited at each site	Descriptive statistics or estimates based on 95% confidence intervals
**Retention**	We will retain 90% of our sample.	• Number of participants that we can gather data from at study end	
**Adherence**	The average proportion of exercise sessions completed will be ≥70%.	• Average proportion of completed exercise sessions	
Secondary			
**Frailty indicators**	Body weight, walking speed, chair stand performance and Four Square Step Test will be responsive to the intervention.<br>Exposure will result in:<br>• maintained or increased body weight;<br>• faster walking speed<br>• less fatigue<br>• improved chair stand scores, Four Square Step test, and grip strength, and increased physical activity level	• Body weight in pounds<br>• 10-metre walk test (seconds<br>• Center for Epidemiologic Studies Depression Scale-fatigue questions<br>• 30 Second Chair Stand (# of stands)<br>• Four Square Step Test (seconds)<br>• Hand dynamometer (in pounds)<br>• Physical Activity Scale (PAS)	*Responsiveness:* standardized response means, or mean change divided by the standard deviation of the changed scores.<br>*Exploratory intention to treat (ITT) and per protocol (PP) analyses:* linear regression[1,2]
**Quality of Life**	Exposure will increase Quality Adjusted Life Years (QALYs)	• EQ5D5L – QALY	*Exploratory ITT and PP analyses:* linear regression[1,2]
**Cost per life-year gained**	No hypothesis at this time	• Questionnaires to assess resource use, documentation of costs of intervention	*Exploratory ITT and PP analyses:* Ratio of incremental costs of intervention & QALY[1,2]
**Protein and energy intake**	Exposure will result in an increase in protein intake	• Nutrient analyses to estimate grams of protein and kilocalories, based on 3-day 24-hour recall at baseline and final visit only	*Exploratory ITT and PP analyses:* linear regression[1,2]
**Participant and provider experiences**	Identify trends and patterns indicating positive experiences and identify needed adaptations	• Exit interviews	Content analyses
**Harms**	Participants will most likely experience minor musculoskeletal adverse events due to the intervention	• Self-report of harms	Descriptive statistics or estimates based on 95% confidence interval

[1] where relevant, adjusted for period, time by exposure interaction & intra-cluster correlation. [2] Sensitivity analyses: with/without caregiver/friend. ITT = intention-to-treat analysis. Based on the initial treatment assignment and not on the treatment eventually received; PP = Per Protocol Analysis. Comparison of treatment groups that includes only those participants who completed the treatment originally allocated.

Table 4: Interview guide to conduct exit interviews with each participant and the exercise professional.

**Predetermined Questions**	
**Questions for the participant:**	**Questions for the kinesiologist:**
Why did you decide to join this study?	Tell me about your experience delivering the MoveStrong program?
What, if any, benefit are you getting/did you get out of your involvement in the study?	What did you like about the instructor manual? What did you dislike about it?
What did you think about the exercise program?	Which exercises did you find more challenging to teach?
What did you think about the nutrition sessions?	How would you feel about delivering the MoveStrong program in future? What might need to change?
Related to your participation in the exercise program, what could we have done better?	How is this program different from your current practice?
What overall changes would you recommend to improve this program?	Can you list certain exercises that participants enjoyed more than other exercises?
	What did you dislike about the exercise program?

REFERENCES

1. Beaudart C, Rizzoli R, Bruyère O, Reginster J, Biver E. Sarcopenia : burden and challenges for public health. 2014;1–8.

2. Lexell J, Taylor CC, Sj M. What is the cause of the ageing atrophy ? Total number , size and proportion of different fiber types studied in whole vastus lateralis muscle from 15- to 83-year-old men. J Neurol Sci. 1988;84:275–94.

3. Cruz-Jentoft A, Baeyens J, Bauer J, Boirie Y, Cederholm T, Landi F, et al. Sarcopenia: European consensus on definition and diagnosis Report of the European Working Group on Sarcopenia in Older People. Age Aging. 2010;39:412–23.

4. Cruz-Jentoft AJ, Landi F, Schneider SM, Zuniga C, Arai H, Boirie Y, et al. Prevalence of and interventions for sarcopenia in ageing adults: a systematic review. Report of the International Sarcopenia Initiative (EWGSOP and IWGS). Age Ageing. 2014 Nov;43(6):748–59.

5. Fried LP, Tangen CM, Walston J, Newman AB, Hirsch C, Gottdiener J, et al. Frailty in Older Adults : Evidence for a Phenotype. J Gerontol. 2001;56A(3):146–57.

6. Yoshimura Y, Wakabayashi H. Interventions for Treating Sarcopenia : A Systematic Review and Meta-Analysis of Randomized Controlled Studies. J Am Med Dir Assoc [Internet]. 2017;18(6):553.e1-553.e16. Available from: http://dx.doi.org/10.1016/j.jamda.2017.03.019

7. Peterson MD, Sen A, Gordon PM. Influence of Resistance Exercise on Lean Body Mass in Aging Adults: A Meta-Analysis. Med Sci Sport Exerc. 2011;43(2):249–58.

8. Wojtek J, Chodzko-Zajko J, Proctor DN, Singh MAF, Minson CT, Nigg CR, et al. Exercise and Physical Activity for Older Adults. Med Sci Sport Exerc. 2009;1510–30.

9. Vlietstra L, Hendrickx W, Waters DL. Review Article Exercise interventions in healthy older adults with sarcopenia : A systematic review and meta-analysis. Aust J Ageing. 2018;37(3):169–83.

10. Liu C-J, Latham NK. Progressive resistance strength training for improving physical function in older adults. Cochrane database Syst Rev. 2009 Jul;(3):CD002759.

11. Sherrington C, Nj F, Gk W, Tiedemann A, Za M, Howard K, et al. Exercise for preventing falls in older people living in the community (Review). Cochrane Database Syst Rev. 2019;(1).

12. Public Health Agency of Canada. How healthy are Canadians? A trend analysis of the health of Canadians from a healthy living and chronic disease perspective. 2001 Annual report. Stress and well-being. Health. 2016.

13. Gibbs JC, McArthur C, Milligan J, Clemson L, Lee L, Boscart VM, et al. Measuring the implementation of a group-based Lifestyle-integrated Functional Exercise (Mi-LiFE) intervention delivered in primary care for older adults aged 75 years or older: a pilot feasibility study protocol. Pilot Feasibility Stud. 2015;1(1):20.

14. Copas AJ, Lewis JJ, Thompson JA, Davey C, Baio G, Hargreaves JR. Designing a stepped wedge trial: three main designs, carry-over effects and randomisation approaches. Trials. 2015 Aug;16(1):352.

15. Skou ST, Roos EM. Good Life with osteoArthritis in Denmark (GLA : D ™): evidence-based education and supervised neuromuscular exercise delivered by certified physiotherapists nationwide. BMC Musculoskelet Disord. 2017;18(72):1–13.

16. Giangregorio L, Papaioannou A, MacIntyre N, Ashe M, Heinonen A, Shipp K, et al. Too Fit To Fracture: exercise recommendations for individuals with osteoporosis or osteoporotic vertebral fracture. Osteoporos Int. 2014;25(3):821–35.

17. Borde R, Hortobagyi T, Granacher U. Dose-Response Relationships of Resistance Training in Healthy Old Adults: A Systematic Review and Meta-Analysis. Sports Med. 2015 Dec;45(12):1693–720.

18. Sherrington C, Michaleff ZA, Fairhall N, Paul SS, Tiedemann A, Whitney J, et al. Exercise to prevent falls in older adults : an updated systematic review and meta-analysis. Br J Sports Med. 2017;51:1749–57.

19. Tricco AC, Thomas SM, Veroniki AA, Hamid JS, Cogo E, Strifler L, et al. Comparisons of Interventions for Preventing Falls in Older Adults A Systematic Review and Meta-analysis. JAMA. 2017;318(17):1687–99.

20. Giangregorio LM, Gibbs JC, Templeton JA, Adachi JD, Ashe MC, Bleakney RR, et al. Build better bones with exercise (B3E Pilot Trial): results of a feasibility study of a multicenter randomized controlled trial of 12 months of home exercise in older women with vertebral fracture. Osteoporos Int. 2018;29(11):2545–56.

21. Fried LP, Tangen CM, Walston J, Newman AB, Hirsch C, Gottdiener J, et al. Frailty in older adults: evidence for a phenotype. J Gerontol A Biol Sci Med Sci. 2001 Mar;56(3):M146-56.

22. Bohannon RW, Andrews AW, Thomas MW. Walking speed: reference values and correlates for older adults. J Orthop Sports Phys Ther. 1996 Aug;24(2):86–90.

23. Radloff LS. The CES-D Scale: A Self-Report Depression Scale for Research in the General Population. Appl Psychol Meas. 1977;1(3):385–401.

24. National Institute for Health Research. Procedure for Measuring HAND GRIP STRENGTH USING THE JAMAR DYNAMOMETER [Internet]. 2016 [cited 2019 May 14]. p. 6. Available from: http://www.uhs.nhs.uk/Media/Southampton-Clinical-Research/Procedures/BRCProcedures/Procedure-for-measuring-gripstrength-using-the-JAMAR-dynamometer.pdf

25. Wong SL. Grip strength reference values for Canadians aged 6 to 79: Canadian Health Measures Survey, 2007 to 2013. Vol. 27, Statistics Canada. 2016.

26. Jones J. A 30-s Chair-Stand Test as a Measure of Lower Body Strength in Community-Residing Older Adults. Res Q Exerc Sport. 1999;70(2):113–9.

27. Moore M, Barker K. The validity and reliability of the four square step test in different

adult populations : a systematic review. Syst Rev. 2017;6(187):1–9.

28. Dite W, Temple VA. A Clinical Test of Stepping and Change of Direction to Identify Multiple Falling Older Adults. Arch Phys Med Rehabil. 2002;83(November):1566–71.

29. Reporting Adverse Reactions to Marketed Health Products: Guidance Document for Industry. 2018.

30. Health Canada. Health Canada Clinical Safety Definitions.

31. Sim J, Lewis M. The size of a pilot study for a clinical trial should be calculated in relation to considerations of precision and efficiency. J Clin Epidemiol [Internet]. 2012;65(3):301–8. Available from: http://dx.doi.org/10.1016/j.jclinepi.2011.07.011

32. Julious SA. Sample size of 12 per group rule of thumb for a pilot study. Pharm Stat. 2005;4:287–91.

33. Marsh AP, Applegate WB, Guralnik JM, Jack Rejeski W, Church TS, Fielding RA, et al. Hospitalizations During a Physical Activity Intervention in Older Adults at Risk of Mobility Disability: Analyses from the Lifestyle Interventions and Independence for Elders Randomized Clinical Trial. J Am Geriatr Soc. 2016 May;64(5):933–43.

34. Gill TM, Pahor M, Guralnik JM, McDermott MM, King AC, Buford TW, et al. Effect of structured physical activity on prevention of serious fall injuries in adults aged 70-89: randomized clinical trial (LIFE Study). BMJ. 2016 Feb;352:i245.

35. Sink KM, Espeland MA, Castro CM, Church T, Cohen R, Dodson JA, et al. Effect of a 24-Month Physical Activity Intervention vs Health Education on Cognitive Outcomes in Sedentary Older Adults: The LIFE Randomized Trial. JAMA. 2015 Aug;314(8):781–90.

36. Iliffe S, Kendrick D, Morris R, Masud T, Gage H, Skelton D, et al. Multicentre cluster randomised trial comparing a community group exercise programme and home-based exercise with usual care for people aged 65 years and over in primary care. Health Technol Assess. 2014;18(49):28.

37. Shubert TE, Smith ML, Goto L, Jiang L, Ory MG. Otago Exercise Program in the United States: Comparison of 2 Implementation Models. Phys Ther. 2017;97(2):187–97.

38. Clemson L, Fiatarone Singh MA, Bundy A, Cumming RG, Manollaras K, O'Loughlin P, et al. Integration of balance and strength training into daily life activity to reduce rate of falls in older people (the LiFE study): Randomised parallel trial. BMJ. 2012;345(7870):1–15.

39. Jansen CP, Nerz C, Kramer F, Labudek S, Klenk J, Dams J, et al. Comparison of a group-delivered and individually delivered lifestyle-integrated functional exercise (LiFE) program in older persons: A randomized noninferiority trial 11 Medical and Health Sciences 1117 Public Health and Health Services. BMC Geriatr. 2018;18(1):1–14.

40. Pavey TG, Anokye N, Taylor AH, Trueman P, Moxham T, Fox KR, et al. The clinical effectiveness and cost- effectiveness of exercise referral schemes : a systematic review and economic evaluation. Health Technol Assess. 2011;15(44).

41. Sørensen JB, Skovgaard T, Puggaard L. Exercise on prescription in general practice : A systematic review. J Prim Health Care. 2006;24:69–74.

42. Martin AM, Woods CB. What Sustains Long-Term Adherence to Structured Physical Activity After a Cardiac Event ? J Aging Phys Act. 2012;20:135–47.

43. Thorpe KE, Zwarenstein M, Oxman AD, Treweek S, Furberg CD, Altman DG, et al. A pragmatic e explanatory continuum indicator summary (PRECIS): a tool to help trial designers. J Clin Epidemiol [Internet]. 2009;62(5):464–75. Available from: http://dx.doi.org/10.1016/j.jclinepi.2008.12.011

44. Beard E, Lewis JJ, Copas A, Davey C, Osrin D, Baio G, et al. Stepped wedge randomised controlled trials : systematic review of studies published between 2010 and 2014. Trials [Internet]. 2015;16(353):1–14. Available from: http://dx.doi.org/10.1186/s13063-015-0839-2

45. Mdege ND, Man M, Taylor CA, Torgerson DJ. There are some circumstances where the stepped-wedge cluster randomized trial is preferable to the alternative : no randomized trial at all . Response to the commentary by Kotz and colleagues. J Clin Epidemiol [Internet]. 2012;65(12):1253–4. Available from: http://dx.doi.org/10.1016/j.jclinepi.2012.06.003
